# Relationship Between Instagram, Body Satisfaction, and Self-Esteem in Early Adulthood

**DOI:** 10.3390/healthcare12212153

**Published:** 2024-10-29

**Authors:** Cristina Flores Mata, Carmina Castellano-Tejedor

**Affiliations:** 1 Faculty of Psychology, Universidad Internacional de la Rioja, 26006 Logroño, Spain; 2 Psynaptic, Psicología y Servicios Científicos y Tecnológicos S.L.P., 08192 Barcelona, Spain; 3 Department of Psychology and Education Sciences, Universitat Oberta de Catalunya (UOC), 08018 Barcelona, Spain; 4 GIES Research Group, Basic Psychology Department, Autonomous University of Barcelona, 08192 Barcelona, Spain; 5 Research Group on Aging, Frailty and Care Transitions in Barcelona (REFiT), Parc Sanitari Pere Virgili & Vall d’Hebron Research Institute (VHIR), 08023 Barcelona, Spain

**Keywords:** body dissatisfaction, self-esteem, Instagram, body image

## Abstract

**Background/Objectives:** This study aimed to explore the effects of Instagram use on body satisfaction and self-esteem in young adults 20 to 40 years (N = 95). Given the widespread use of social media and its potential influence on body image, we sought to understand how Instagram use may contribute to body dissatisfaction and self-esteem, particularly through quantitative analysis of self-report measures. **Methods:** A cross-sectional survey design in which the Rosenberg Self-Esteem Scale (RSES), the Body Shape Questionnaire (BSQ), and additional ad hoc questions designed to assess Instagram usage patterns were employed. **Results:** The results indicated that greater Instagram use is associated with increased body dissatisfaction (*p* = 0.005), although it did not significantly affect self-esteem (*p* = 0.211). Gender did not play a significant role in these relationships (*p* = 0.173). Notably, a significant positive correlation was found between body satisfaction and self-esteem, showing that individuals with higher body satisfaction also reported higher self-esteem (*p* < 0.001). Further analyses indicated that users exposed to appearance-centered content were more likely to report body dissatisfaction. **Conclusions:** These findings suggest that Instagram usage, particularly in the context of appearance-focused content, has a considerable impact on body dissatisfaction among young adults but does not appear to influence self-esteem. This highlights the importance of developing interventions focused on promoting healthy social media habits and critical content engagement to mitigate negative impacts on body image. Social media exposure should be a key component in future interventions designed to improve body image and overall psychological well-being.

## 1. Introduction

The multidimensional paradigm shift brought about by social networks has altered, among many other aspects, individuals’ relationship with themselves concerning body satisfaction and self-esteem. Numerous studies have shown that constant exposure to social networks like Instagram, which bombards its users with images of perfect and idealized bodies on a daily basis, promotes physical comparison behaviors [1], body dissatisfaction [2,3], and low self-esteem [4] due to the pressure generated by unattainable beauty standards that have evolved significantly in recent years and are now greatly influenced by social networks [5]. Instagram’s unique emphasis on visual content creates distinct patterns of social comparison and body image concerns that differ from other platforms. Body dissatisfaction, as well as damaged self-esteem, increase the likelihood of suffering from an eating disorder [6,7] or disorders such as muscle dysmorphia or body dysmorphia [3], as well as presenting low emotion regulation [8], depression, or anxiety [9,10]. Additionally, social network addiction increases body dissatisfaction and predisposes one to low self-esteem [3]. For all these reasons, this research is relevant, as it aims to analyze how exposure to Instagram is a risk factor for body satisfaction and self-esteem in early adulthood, as well as to establish gender differences, given that most studies in this area are limited and scarce.

### 1.1. Self-Esteem: Conceptualization

Self-esteem is a fundamental concept, defined as the subjective appreciation that a person makes of their own worth, influenced by their social environment and personal experiences. Rosenberg [11] defines self-esteem as “the positive evaluation an individual makes of themselves in terms of respect and worth” (p. 30) [11].

Abdel-Khalek [12] defines it simply as general self-satisfaction, while Branden [13] emphasizes its role in preparing individuals for life’s challenges based on their confidence and emotional responses to various aspects of their identity and abilities. Self-esteem is multidimensional, affecting various life areas and evolving significantly during key developmental stages, such as adolescence [12]. This period is particularly critical due to significant psychological changes and identity integration, heavily influenced by family, teachers, and peers. Self-esteem is a global evaluation of one’s worth, encompassing beliefs, emotions, competencies, and the perceived affection one deserves, ranging from high to low based on the outcome of this evaluative process [12].

### 1.2. Low Self-Esteem as a Risk Factor

The study of the relationship between self-esteem and gender has been extensive and diverse. The literature states that men tend to have better self-esteem than women, seeing themselves as more valuable and skilled. This trend begins in adolescence and persists until adulthood, balancing out once old age is reached. These differences are due to both sociocultural contexts and genetic and biological processes that go beyond culture [14,15].

People with low self-esteem feel inferior, less important, and less competent, suffer from greater emotional instability, and are more likely to associate with people with low self-esteem, feeling irritated in the presence of people with higher self-esteem [12,13]. Regarding technology use, Casale et al. [16] state that low self-esteem increases smartphone dependency, impulsivity, sensation seeking, and substance use. Liu et al. [17] have evidenced that low self-esteem is a risk factor for the development of mental health problems such as depression, anxiety, or eating disorders, being related to academic stress and suicidal ideation [18], as well as a reduced capacity to cope with problems [12] and a higher likelihood of experiencing burnout syndrome [19].

### 1.3. Body Satisfaction: Conceptualization

We live in a body-worshiping society. We are surrounded by stimuli that exalt and idealize physical appearance, from media, social networks, the fashion industry, cinema, advertising, and toys to the amount of money and effort invested in improving appearance through diets, physical exercise, and cosmetic enhancements. These factors influence body image, and more specifically, satisfaction with physical appearance [20]. To understand body satisfaction, Taylor [21] emphasizes that it is essential to distinguish between how an individual perceives their body size and the concern they feel about their body shape, as these are not equivalent terms and might not be linked. A person can misjudge how they perceive themselves physically and not feel dissatisfaction with their image and vice versa [21]. Once this distinction is made, body satisfaction can be understood as a person’s contentment with various parts of their body or their overall appearance, as well as the comfort they feel with their body in different contexts [22]. Body image satisfaction is a construct closely related to general self-esteem. Authors affirm that women are the most dissatisfied and concerned about their figure, weight, or physical shape [20,23]. However, authors like Baile [24] have shown that gender differences are decreasing, as men also suffer from body image issues, specifically tending to worry more about their muscle mass and being toned and defined. Nevertheless, no significant gender differences were found in how social networks impact body dissatisfaction [25]. Saiphoo and Vahedi [26] state that there is a significant relationship between body dissatisfaction and heavy social media use. Tiggemann et al. [20] highlight that Instagram use increases women’s dissatisfaction with their facial features. Lastly, Lowe and Grieve [27] warn about the dangers of following many fashion influencers, as this can negatively affect users’ body satisfaction.

### 1.4. Distorted Perception of Body Image

The construction of body image encompasses perceptual, cognitive, affective, and behavioral aspects [28,29]. The perceptual aspect influences our body satisfaction [30] and refers to how accurately an individual perceives their body size, shape, or weight. Body image is said to be distorted when an individual’s evaluation of their image leads to overestimating or underestimating their appearance disproportionately [31]. This occurs when there is a significant difference between a person’s actual physical size or shape and their self-evaluation or subjective judgment [32]. Distorted body image can affect physical and psychological health, damaging self-esteem, mood, sense of competence, or social functioning [29], and can even precipitate the development of mental health disorders such as eating disorders or body dysmorphic disorders [33]. In individuals with anorexia, there is perceptual distortion regarding weight or body constitution, as they tend to evaluate themselves as heavier than they actually are. In body dysmorphic disorders, there is significant distress due to perceived physical imperfections that are not noticeable to others. Individuals with muscle dysmorphia show excessive concern about their muscle mass, perceiving themselves as thinner and weaker than they are [33]. The association between social media exposure and distorted body image has been scarcely explored in scientific studies, indicating a need for more research in this specific area. However, Parrillo and Troncoso [34] found significant results regarding Instagram use and its impact on body perception.

### 1.5. Body Dissatisfaction as a Risk Factor

Those who feel significantly dissatisfied with their physical appearance are more predisposed to engage in strict diets, follow intense exercise routines, undergo surgeries to alter their appearance, and consequently develop mental health issues such as eating disorders, body dysmorphic disorder, or muscle dysmorphia [24,33]. Additionally, stress, low self-esteem, and experiences of bullying have also been shown to trigger body dissatisfaction issues [35]. Exposure to social networks is a risk factor for developing body dissatisfaction, which in turn contributes to a higher risk of developing eating disorders [36]. Romano et al. [37] determined that body dissatisfaction correlates with an increase in binge eating, purging, restriction, excessive exercise, and behaviors aimed at increasing muscle mass. Furthermore, comments about physical appearance from parents during childhood and adolescence are related to greater body dissatisfaction [38]. Dissatisfaction increases the likelihood of experiencing obsessive concern about one or more body parts, as well as paying excessive attention to areas considered defective. This symptomatology is exacerbated if users physically compare themselves with photos of other profiles on social networks [3]. Regarding muscle dysmorphia, the likelihood of developing it increases with greater physical or muscular dissatisfaction [39]. Some of the most common symptoms include concern about not being sufficiently muscular, avoiding social situations where they have to expose or show their physique, continuous physical checks in mirrors or shop windows, rigid and strict dietary behaviors, compulsive physical exercise, low self-esteem, social comparison, body image distortion, and impact on work, social, or personal life, among other aspects [24,40]. These symptoms are intensified at higher levels of body dissatisfaction [24].

### 1.6. Instagram and Its Impact on Body Image

Instagram is a platform dominated by content related to physical appearance. Users of this platform tend to feel more dissatisfied with their body image, initiate dieting behaviors, restrict food, binge eat, have worse self-talk regarding their image, seek more external approval, and engage in higher levels of social comparison [41]. Additionally, addiction to physical exercise, anxiety, and depression related to body image are magnified; there is an increase in the use of pharmacological substances or anabolic steroids and a significant decrease in the quality of life of individuals [42]. Yang et al. [43] describe that continuous use of social media and constant exposure to images of normative physiques that reinforce the internalization of beauty standards are associated with a decrease in body self-esteem. This can lead to concern about external evaluation, body dissatisfaction, and an increase in physical comparison. It has been demonstrated that high exposure to social media increases the development of eating disorders and body dysmorphia [44]. Furthermore, social media use is related to feelings of loneliness, low self-esteem, and low body self-esteem [45]. Vuong et al. [46] state that social media expands the thin and muscular beauty ideal. The integration of this beauty ideal correlates with body dissatisfaction in both genders equally [25,46] The widespread beauty canon exalts perfect and unattainable shapes and figures; this perfectionism propagated on social media is a significant risk factor in the development of dysmorphic disorders [47]. The motivations for body exposure on Instagram are driven by the platform’s visual nature and the pursuit of social validation, which aligns with Vuong et al. [46]. Instagram, as a predominantly visual platform, encourages users to share images and videos that highlight their physical appearance. This emphasis on visual content fosters an environment where physical attractiveness becomes a key currency for gaining attention and approval from peers. According to Vuong et al. [46], users are often motivated by the desire for social validation, seeking likes, comments, and followers to enhance their social standing and self-worth. This behavior is reinforced by Instagram’s algorithm, which promotes visually appealing content, further incentivizing users to conform to beauty standards and engage in body exposure. The platform’s design thus creates a feedback loop where visual self-presentation and social validation are intertwined, leading individuals to prioritize appearance over other attributes. Additionally, research has shown that this constant need for validation can exacerbate body image concerns and contribute to a cycle of self-comparison and dissatisfaction, as users continuously measure their own appearance against the curated images of others [20].

### 1.7. Sociocultural Factors

Vuong et al. [46] clarify that the sociocultural context is fundamental to understanding body dissatisfaction. The cultural macrosystem surrounding an individual involves beauty standards, ethnicity or culture, and the influence of the media. The meso and microsystems involve peers, family, education, and personal experiences. All these factors form the Tripartite Influence Model [48], which describes these factors as pathways through which we internalize and reinforce beauty ideals. The beauty canon has evolved over the years. Currently, the male ideal is characterized by the pursuit of a muscular and lean body (broad back, wide shoulders, prominent abdominal muscles, and a narrow waist and hips). However, the female beauty ideal predominantly desires thinness, although muscularity is increasingly sought after in women [24,49]. Both female and male bodies strive to meet indicators of youth and have tanned skin [49]. Additionally, shapes and sizes tend to be sexualized, especially in girls, highlighting full lips, voluptuous breasts and buttocks, and a small waist [50]. Since these factors are practically unattainable in a natural and healthy way, both men and women become disappointed and frustrated, feeling dissatisfied and more likely to resort to unhealthy behaviors to alleviate this dissatisfaction, such as obsessive dieting, excessive exercise, or the use of anabolic substances [49]. Exposure to social networks, media, cinema, advertising campaigns, the fashion industry, family, and close surroundings act as transmitters and maintainers of the beauty canon, leading to frequent comparison behaviors with other physiques [49], severely affecting self-esteem and body satisfaction [51].

### 1.8. Personal Factors

Personality traits, low self-esteem, experiences of bullying, low emotional intelligence, life stage, and even gender have been closely linked to body dissatisfaction. Regarding personality traits, those that predispose individuals to greater body dissatisfaction include high scores in neuroticism and low levels of extraversion and conscientiousness [52], as well as low self-esteem [9]. Internal dialogue acts as a mediator between thoughts, emotions, and behavior and the perception of one’s body image as valuable. A healthy internal dialogue can facilitate the development of strategies to manage anxiety, sadness, anger, dissatisfaction, or guilt that may arise from not feeling comfortable with one’s appearance [53]. In fact, emotional intelligence reduces the likelihood of feeling dissatisfied with physical appearance [54]. Moreover, traumatic life experiences, such as bullying or other forms of severe humiliation, can trigger higher levels of body dissatisfaction [35,55]. The most vulnerable life stage is adolescence, as it corresponds to the formation of identity [54,55]. However, few studies have investigated early adulthood concerning body image. Quittkat et al.’s [56] meta-analysis revealed that women tend to feel more dissatisfied with their image as they age, while men typically place more importance on their physique when they are young but become more dissatisfied as they grow older. Lastly, the idea that women are more affected by body dissatisfaction has long been acknowledged [23,56,57]. However, gender differences are diminishing, especially concerning muscularity and leanness, as opposed to thinness [24].

### 1.9. Objectives and Hypothesis

This study contributes to understanding how social media platforms influence psychological well-being and highlights the need for targeted interventions. The objectives of this study are to analyze the impact of Instagram on body satisfaction and self-esteem, considering gender differences. We hypothesize that more Instagram use correlates with greater body dissatisfaction (H1) and lower self-esteem (H2) and that these effects differ by gender (H3 and H4).

## 2. Materials and Methods

### 2.1. Design

Our research follows a cross-sectional survey design with a non-probabilistic general population sample [58]. We collected quantitative data from a general sample at a single point in time through an online questionnaire, without conducting follow-up over time.

### 2.2. Participants

Participants were selected using a non-random, purposive convenience sampling method, which was deemed appropriate given the study’s focus and objectives. We employed a snowball recruitment strategy, where initial participants referred close contacts who met the inclusion criteria. The final sample comprised 95 participants, selected based on the following inclusion criteria: (1) individuals aged between 20 and 40 years (inclusive), (2) possession of a smartphone, (3) active Instagram use, and (4) fluency in Spanish. Exclusion criteria included (1) individuals under the age of 18 and (2) individuals currently diagnosed with an eating disorder.

### 2.3. Study Measurement Tools

The instruments used to measure the study variables were as follows:

- **Body Shape Questionnaire (BSQ):** Developed by Cooper et al. [22] and adapted into Spanish by Raich et al. [59], this questionnaire measures dissatisfaction with body image, as well as concern about one’s own image and self-perception. The BSQ consists of 34 items evaluated on a 6-category Likert-type scale (*Never, Rarely, Sometimes, Often, Very Often, Always*). It assesses body image satisfaction and dissatisfaction, the frequency and type of thoughts about physical appearance, social comparisons, feelings about one’s own physique, and compensatory behaviors to regulate anxiety generated by body image dissatisfaction (e.g., Item 9: “*Has being with thin men/women made you feel self-conscious about your shape?*” or item 12: “*Have you noticed the shape of other men and felt that your shape compared unfavorably?*”). The BSQ scores are obtained by summing the total scores, with a minimum of 34 points (high body satisfaction) and a maximum of 204 points (high body dissatisfaction). Neither Cooper et al. [22] nor other researchers have established universal benchmarks; thus, this study established the following categorical ranges: high satisfaction (34–76), moderate satisfaction (77–119), low satisfaction (120–160), and very low satisfaction (161–204).
- **Rosenberg Self-Esteem Scale:** Developed by Rosenberg [11] and adapted into Spanish by Atienza et al. [60], this scale measures the subjective evaluation of one’s own self-esteem in terms of self-respect and self-worth (e.g., item 1: “*On the whole, I am satisfied with myself*” or item 7: “*I feel that I’m a person of worth*”). The Rosenberg Self-Esteem Scale has 10 items evaluated on a 4-category Likert-type scale (*Strongly Disagree, Disagree, Agree, Strongly Agree*). It assesses thoughts, beliefs, and feelings about one’s abilities, value, and competence. The results are interpreted considering the following categorical groups: low self-esteem (10–15), medium-low self-esteem (16–21), medium self-esteem (22–27), high self-esteem (28–33), and very high self-esteem (34–40).
- **Single-Item Self-Esteem Scale:** Developed by Trzesniewski [61] and translated into Spanish for this study, this scale measures the overall perceived level of self-esteem of the individual. The Single-Item Self-Esteem Scale consists of one item, “*I have high self-esteem in general*”, evaluated on a Likert scale with numerical categories from 1 to 7, where 1 is “*Not very true of me*” and 7 is “*Very true of me*”. This scale does not have specific cutoff points, as it is a subjective and simplified assessment of self-esteem. Therefore, the results are interpreted according to numerical categories: intervals from 1 to 3 approximately correspond to low self-esteem perception, intervals 3–4 correspond to moderate self-esteem, and intervals 5–7 correspond to high self-esteem.
- **Ad Hoc Interest Questions:** These questions were developed to complement the necessary information for this study. The selected questions gather information about physical comparison behaviors, the impact of Instagram on self-esteem and satisfaction, concern about one’s image and figure, concern about musculature, the influence of sociocultural factors on satisfaction and self-esteem, and cognitive, emotional, and behavioral aspects related to body image, among other issues. The 8 items with closed response options related to Instagram use were the following:
  1. How often do you use Instagram per week?
  2. What is your daily average total time spent on Instagram? (You can check it in the app under “Your Activity—Time on Instagram” for more accuracy)
  3. What is the purpose of your Instagram account(s)?
  4. Select the types of accounts you follow or are most interested in when using Instagram.
  5. How often do you compare your photos with those of others on Instagram?
  6. Have you taken specific measures to change your appearance or lifestyle as a result of these comparisons on Instagram?
  7. Have you considered unfollowing accounts that make you feel bad about yourself due to comparisons?
  8. What strategies do you use to maintain a positive and healthy attitude towards comparison on Instagram?

Instagram use was measured quantitatively (hours per day/week spent on the platform). Additional qualitative measures (purpose of use, type of accounts followed) were also noted. The use of quantitative measures in our study allows for a broad assessment of trends and patterns, providing a comprehensive overview of the relationships between Instagram use, body satisfaction, and self-esteem. Quantitative methods enable us to analyze large datasets and identify statistically significant correlations and differences, which are essential for drawing generalizable conclusions.

The reliability of the scales was assessed using Cronbach’s alpha, with results indicating acceptable reliability for both the self-esteem scale (α = 0.85) and the body satisfaction scale (α = 0.88). Additionally, split-half reliability was calculated, yielding a coefficient of 0.82 for the self-esteem scale and 0.84 for the body satisfaction scale. Validity indices, including content and construct validity, were also evaluated, demonstrating strong alignment with theoretical expectations and consistent factor structures.

### 2.4. Procedure

To collect the data of interest, we created an online questionnaire using the Google Forms platform. The primary objectives of this survey were to gather information about the participants’ body satisfaction and self-esteem. The previously mentioned instruments and tests were used to achieve these objectives. The questionnaire was primarily distributed to attendees of a psychoeducational workshop on body image, and they were asked to share the form link with friends or family members who met the study’s inclusion criteria. The introductory part of the form explains the purpose of this study. To complete the form, participants must read and give their consent through a confidential document. The first section of the form corresponds to sociodemographic data. Next, participants must fill out the Instagram activity section, which includes questions related to their use of the platform. Following this, the self-esteem section contains questions related to the participants’ subjective perception of their self-esteem. Finally, the body satisfaction section includes questions associated with the satisfaction and dissatisfaction generated by their physical appearance. The form takes approximately 12 to 15 min to complete, and the responses were collected in an Excel document for subsequent analysis.

### 2.5. Data Analysis

The statistical program SPSS Statistics version 29.0 for Windows was used for data analysis. First, descriptive statistics were performed for the sample description variables and sociodemographic factors. Specifically, the mean, standard deviation, and minimum and maximum ranges of the quantitative variables were calculated, and frequencies and percentages were calculated for the categorical variables. Subsequently, the Mann–Whitney U test and Kruskal–Wallis H test were applied to the sociodemographic variables and the explanatory factors of the self-esteem and body satisfaction variables. The reliability of the BSQ and the Rosenberg Self-Esteem Scale in their application to the sample was determined using Cronbach’s alpha coefficient. Secondly, an exhaustive analysis of the study variables (self-esteem, Instagram use, and body satisfaction) was conducted. The mean, standard deviation, and minimum and maximum ranges, as well as frequencies and percentages, were calculated. To test the hypotheses, the Kolmogorov–Smirnov test was first performed on the variables of Self-Esteem (Rosenberg Self-Esteem Scale), Body Satisfaction (BSQ), and Instagram Use. The assumption of normal distribution for all variables was rejected (*p*-value < 0.05). Consequently, non-parametric tests were applied—the Mann–Whitney U test, the Kruskal–Wallis H test, and the Spearman correlation test—depending on the type of analysis and variables to be studied. According to the Spearman correlation test, the following correlation ranges were considered to interpret the correlation strength between variables: weak (<0.3), moderate (0.4–0.6), and high (>0.6). In cases where the data followed a normal distribution (new aggrupation of the sample), the ANOVA test was used. Specifically, ANOVA was employed to compare differences in Instagram usage between groups with high–moderate body satisfaction and those with low–very low body satisfaction. Statistical significance was considered with a *p*-value < 0.005 (*p* < 0.005, 95% CI).

## 3. Results

### 3.1. Sample Description

The total number of participants was 104, of which 9 were excluded for not meeting the inclusion criteria, resulting in a final study sample of 95 participants. The mean age was 25.83 (*SD* = 4.25) within a range of 20–39 years. Of these, regarding to gender, 25.3% (*n* = 24) were men, 73.7% (*n* = 70) were women, and 1.1% (*n* = 1) were non-binary individuals. Detailed information on the educational level, nationality, place of residence, profession, history of eating disorders, frequency of comparison, time spent thinking about defects, purpose of the Instagram account, and modifications in appearance or lifestyle is presented in Table 1. No missing data were observed in the sample; therefore, Table 1 does not include any notation for missing data.

### 3.2. Explanatory Factors Associated with Self-Esteem and Body Satisfaction

To understand the differences in self-esteem and body satisfaction scores according to explanatory factors such as sociodemographic data (age, gender, nationality, place of residence, educational level, profession, and history of past eating disorders), frequency of comparison, time spent thinking about physical defects, and purpose of the Instagram account, as well as modifications in appearance or lifestyle, a series of non-parametric statistical tests were conducted, specifically the Mann–Whitney U test and the Kruskal–Wallis H test. The variables that were statistically significant are presented in Table 2. Only significant results are reported in Table 2.

### 3.3. Results Related to Body Satisfaction of the Sample

The mean total score of the sample for this instrument was 82.45 (*SD* = 32.40, range 34–204), placing it in a moderate range (77–119). The detailed results for the total sample, according to the BSQ interpretation, are presented in Table 3. Only significant results are reported in Table 3.

The non-parametric Mann–Whitney U test was applied, which proved to be non-significant (*p* = 0.173), indicating that gender does not appear to influence the variable of satisfaction.

### 3.4. Relationship Between Instagram Use and Body Satisfaction of the Sample

To assess the correlation between Instagram use and body satisfaction, the Spearman correlation test was applied to the data collected from the total sample. The results showed a weak positive correlation between the two variables (r = 0.285, *p* = 0.005), suggesting that as Instagram use increases, body dissatisfaction also increases.

Furthermore, the overall sample was divided into two groups. The first group (*n* = 71) consisted of users with high–moderate body satisfaction, and the second group (*n* = 24) included users with low–very low body satisfaction. An analysis of variance (ANOVA) was then conducted to examine the differences between groups regarding Instagram use. The results revealed significant differences between groups (*p* = 0.035, F = 4.580). These findings indicate that the level of body dissatisfaction varies according to differences in platform use, with those who use Instagram more (>3 h) showing worse body satisfaction compared to those who use Instagram less (<1 h).

### 3.5. Results Related to Self-Esteem of the Sample

The mean score of the sample was 30.06 (*SD* = 5.56). Most of the sample (*n* = 50) falls within the very high self-esteem range (34–40), followed by a smaller group (*n* = 20) in the low self-esteem range (10–15). The results for the total sample, according to the interpretation of the Rosenberg Self-Esteem Scale (1965), are detailed in Table 4. Only significant results are reported in Table 4.

The non-parametric Mann–Whitney U test was applied, and the findings showed no significant differences between gender and self-esteem levels (*p* = 0.463).

### 3.6. Relationship Between Instagram Use and Self-Esteem of the Sample

A Spearman correlation test was conducted to analyze the relationship between Instagram use and participants’ self-esteem. Interestingly, no significant correlation was found between Instagram use and self-esteem (r = 0.129, *p* = 0.211). This suggests that other factors, such as individual differences in social media engagement or the specific type of content consumed, may play a role in mediating this relationship.

Participants reported a range of experiences with Instagram, from feeling inspired and motivated by fitness influencers to experiencing anxiety and pressure to conform to beauty standards. These qualitative insights provide a deeper understanding of the personal impacts of Instagram use.

The low significant relationship between these variables should not be taken as sufficient reason to dismiss the association between Instagram use and self-esteem levels. Therefore, the overall sample was divided into two subgroups: one group (*n* = 71) included participants with medium–high, high, and very high self-esteem, and the second group (*n* = 24) comprised users with medium-low and low self-esteem. An analysis of variance (ANOVA) was then performed to examine the differences between groups regarding Instagram use. In this case, the results did not show statistically significant relationships between higher or lower Instagram use and self-esteem levels based on the comparison groups (*p* = 0.656).

### 3.7. Relationship Between Body Satisfaction and Self-Esteem of the Sample

A Spearman correlation test was applied to the variables of self-esteem and body satisfaction to analyze the relationship between them. The findings revealed a moderate negative significant correlation (r = −0.457, *p* < 0.001). This indicates that as self-esteem levels increase, body dissatisfaction decreases and vice versa.

## 4. Discussion

The general objective of this project was to analyze the impact of Instagram use on the level of body satisfaction and self-esteem in young adults aged 20 to 40 years. Additionally, this study aimed to understand the relationship between body satisfaction and self-esteem within the sample, as well as determine the variability in self-esteem and body satisfaction levels according to gender.

After administering an online questionnaire and scoring the evaluation instruments—the BSQ and the Rosenberg Self-Esteem Scale—a data analysis was performed based on the sample results, which followed a non-normal distribution.

To begin, several explanatory factors were considered that presented significant differences in the levels of self-esteem and body satisfaction in the study sample. First, differences in participants’ professional choices explain the variability in self-esteem scores. Individuals diagnosed with an eating disorder in the past seem to show significantly different results in both self-esteem and body satisfaction compared to those without such a diagnosis. As Liu et al. [17] established, low self-esteem is a risk factor for developing an eating disorder, and, similarly, body dissatisfaction serves as both a cause and consequence of eating disorders [24,33]. Therefore, it is not surprising that a history of such disorders explains differences in current levels of self-esteem and body satisfaction. Other explanatory factors analyzed included the frequency with which participants compare themselves to others on Instagram [61], which aligns with the research of Yang [43] and Ryding and Kuss [3], the time spent thinking about physical defects, and the tendency to modify appearance or lifestyle, which significantly impact fluctuations in self-esteem and body satisfaction scores within the sample. Furthermore, recent studies suggest that social comparisons on platforms like Instagram are linked to negative body image and reduced self-esteem, particularly during the COVID-19 pandemic, when social media use increased [62]. These findings are consistent with our results, highlighting the need for targeted interventions focused on social media literacy and self-esteem enhancement. Finally, how participants perceive their overall level of self-esteem and body satisfaction also influences the scores of each variable. Contrary to theory, in the studied sample, factors such as age, gender, nationality, place of residence, educational level, and the purpose of the Instagram account did not significantly influence the variability in self-esteem and body satisfaction scores. Unexpectedly, the lack of a significant relationship between Instagram use and self-esteem suggests potential moderating variables, such as individual differences in social media engagement. Studies have shown that certain activities on social media, such as selfie engagement, are associated with increased body objectification and lower self-esteem, further complicating the relationship between social media use and self-esteem [61,63].

Regarding the first hypothesis (H1), which posited that a greater number of hours spent on Instagram would correlate with higher levels of body dissatisfaction, sufficient statistical evidence was found to accept this hypothesis. However, this correlation is weak (<0.3), which may be related to the small sample size or the limited number of study variables, so these conclusions should be interpreted with caution. Nonetheless, the study results align with the established scientific literature by authors such as Uchôa et al. [36] or Rounsefell et al. [41], who assert that exposure to social networks is a risk factor for feeling dissatisfied with one’s figure. Similarly, statistically significant differences were also found regarding Instagram use between subgroups—people with high–moderate body satisfaction and people with low–very low body satisfaction—thereby fully supporting H1. Participants reported viewing a variety of content, predominantly focused on fitness and lifestyle influencers, which, despite these results, was expected to influence their body image perceptions. Recent studies have highlighted the significant impact of social media, particularly platforms like Instagram, on users’ self-esteem and body image. For example, Vogel et al. [62] conducted a longitudinal study that found that an increased use of social media during the COVID-19 pandemic was associated with lower self-esteem and greater body dissatisfaction among adolescents. This study emphasizes that frequent social media use, particularly in times of crisis, can exacerbate negative self-perceptions and increase the risk of mental health issues. These findings align with this current study’s results, suggesting that the more time individuals spend on Instagram, the more likely they are to experience decreased body satisfaction. This evidence supports the need for targeted interventions aimed at promoting healthier social media habits and improving digital literacy to mitigate the negative effects on self-esteem and body image.

As for the second hypothesis (H2), which stated that a greater number of hours spent on Instagram would correlate with higher levels of low self-esteem, no sufficient statistical results were found in the sample to accept it, and it had to be rejected. These results differ from studies such as those by Rizwan et al. [44] or Pop et al. [45], which demonstrated a close association between Instagram use and low self-esteem. The observed lack of significant correlation between Instagram use and self-esteem may be influenced by confounding variables such as baseline self-esteem levels, offline social support systems, and the nature of Instagram interactions. Furthermore, the Fear of Missing Out (FoMO), which has been heightened by social media, can also negatively impact self-esteem and increase anxiety, as highlighted in recent studies [64]. Future research should explore these factors in greater detail to elucidate their roles.

The third hypothesis (H3) proposed that users with high self-esteem would show greater body satisfaction, while users with low self-esteem would show lower body satisfaction. The sample results are sufficient to accept this hypothesis, which aligns with scientific studies by authors such as Baile [24], who establish the relationship between low self-esteem and body dissatisfaction. These results coincide with Liu et al. [17], who state that high self-esteem is related to a better self-view in terms of abilities, skills, and better body perception. On the other hand, Yang et al. [43] determined the relationship between low body self-esteem and poorer body image satisfaction. Our findings also align with those of Ryding and Kuss [3], who also observed a significant impact of social media on body dissatisfaction but not on self-esteem. However, our results diverge from those of Rizwan et al. [44], who found a strong correlation between social media use and low self-esteem, highlighting the need for further investigation into contextual factors.

The fourth hypothesis (H4) posited that body dissatisfaction is significantly higher in females than in males. However, after analyzing the sample results, no significant differences were found, leading to the rejection of the fourth hypothesis, contrary to what was established by Cash and Smolak [65], who postulated differences in body dissatisfaction levels by gender. Other authors specify an increase in body dissatisfaction among women [20,23,57]. However, Marques et al. [25] did not find substantial differences in body dissatisfaction based on gender.

Finally, the fifth hypothesis (H5) proposed that low self-esteem is significantly higher in females than in males. Based on the study results, this hypothesis is rejected. Nonetheless, studies by authors such as Casale [16] and Josephs et al. [15] indicate that men show a greater tendency than women to have higher self-esteem. Therefore, even though the hypothesis is rejected, the results coincide with findings from other research [2,66,67,68,69].

Our results align with those of Scully et al. [1] regarding social media’s impact on body image. Novel findings include the weak correlation between Instagram use and self-esteem, suggesting other mediating factors.

### 4.1. Limitations

One of the main limitations of this study was its sample size, as valid information was obtained from only 95 participants. For studies of this type, it would be advisable to use larger samples with a normal data distribution, which would allow for the application of more powerful statistical tests. Also, the purposive sample from a body image workshop might have limited generalizability. The selected sample, consisting of individuals actively engaged in body image discussions, offers valuable insights into a population that aligns closely with the study’s objectives. This approach effectively targets a group with shared characteristics relevant to the research questions, as demonstrated in previous studies employing similar methods [70,71]. By utilizing a convenience sample, this study increased the likelihood of recruiting participants deeply involved in discussions about body image—an essential factor for the research. Although convenience sampling limits generalizability, it was appropriate for the exploratory nature of this study, which sought to examine specific behaviors within a relevant subgroup [72]. Focusing on individuals already engaged in body image discussions allows for a more nuanced exploration of the dynamics between Instagram use, body satisfaction, and self-esteem. This targeted approach enables a deeper understanding of how these phenomena impact a population particularly vulnerable to body image concerns. However, we acknowledge that this sampling method has limitations, particularly in reducing the ability to generalize findings to a broader population. Future research should consider more diverse sampling methods to mitigate this bias and provide a more representative perspective. Previous studies in the literature also caution against the risks of using homogenous groups when studying socially sensitive topics [73,74]. Future studies should include more diverse groups. Another limitation was the gender imbalance, with 73.7% of the sample being women and 25.3% being men. This imbalance might have influenced and biased the interpretation of the results. We acknowledge that our sample was drawn from a specific group of individuals who attended a psychoeducational workshop on body image. While this may appear to limit the generalizability of our findings, there are several methodological benefits to this approach. A homogeneous sample group enhanced the internal validity of this study by controlling for extraneous variables that could confound the results, allowing for a more precise examination of the relationships between Instagram use, body satisfaction, and self-esteem within a group actively engaged in body image discussions. The selected sample is particularly relevant to the research questions, as individuals who attend body image workshops are likely to have heightened awareness and concern about body image issues, making them an ideal population for studying the impact of social media on these constructs. Furthermore, by using well-established instruments, we can compare our findings with normative data from broader populations, helping to contextualize our results within the larger body of research on body image and self-esteem [11,22]. Another limitation was the use of a few standardized instruments with well-defined correction scales, as well as the development of ad hoc questions that lack reliability and validity. We also recognize that the original question used to measure Instagram use in the study was not sufficient to fully capture screen time and its potential effects on hyper connection. Initially, the questionnaire asked participants, “How often do you use Instagram per week?” However, this question did not provide enough detail to accurately assess the extent of Instagram use. To address this limitation, future iterations of the questionnaire will include more detailed questions, such as “What is your average daily time spent on Instagram?” This change will allow for a more accurate measurement of screen time and hyper connection, providing a better understanding of the relationship between Instagram use and variables like self-esteem and body satisfaction. Additionally, some variables that could have been explored in greater depth (e.g., profession) were selected without analyzing aspects related to different professions (e.g., professions related to image, body, sports centers, fashion, etc.). Finally, we cannot rule out the possibility that participants committed social desirability bias when answering the questions, as a significant percentage of the responses obtained differ significantly from what is indicated in the scientific literature (e.g., when asking about the time spent on social media or questions related to their self-esteem and body satisfaction).

### 4.2. Prospective Directions

We have outlined several avenues for future research to address the issues identified in this project. Therefore, for future studies, we propose the following:

- Expand and balance the sample: Increase the sample size and ensure gender balance to allow for the application of more robust and representative statistical tests.
- Utilize updated instruments: Employ a variety of contemporary instruments to detect social desirability and body dysmorphia, in addition to collecting detailed data on Instagram usage and its impact on users.
- Conduct longitudinal studies: Implement a longitudinal study design to examine changes over time in Instagram usage and its effects on self-esteem and body satisfaction.
- Include control and experimental groups: Consider the inclusion of control and experimental groups and stratify the analysis by gender and age ranges.
- Investigate the impact on eating and dysmorphic disorders: Explore the impact of Instagram on eating disorders and body dysmorphic disorders, given the links between these disorders and self-esteem.
- Develop intervention programs: Create intervention programs aimed at addressing issues of low self-esteem, body dissatisfaction, and social media addiction to mitigate the associated distress.

These recommendations are intended to enhance the rigor and depth of future research in this area, ultimately leading to more comprehensive and actionable insights.

## 5. Conclusions

The primary objective of this research was to analyze the relationship between Instagram use and the levels of self-esteem and body satisfaction in young adults aged 20 to 40 years. Additionally, this study evaluated the impact of gender, sociodemographic factors, and other explanatory variables on these relationships.

The findings suggest that the number of hours spent on Instagram is significantly correlated with increased levels of body dissatisfaction, indicating that prolonged use of the platform may be a risk factor for negative body image. By contrast, the data did not provide strong evidence that Instagram use directly impacts self-esteem levels, suggesting that other moderating factors may play a role. This underscores the importance of understanding the different dimensions of social media use and its varied impacts on psychological well-being.

Moreover, this study observed that individuals with higher self-esteem tend to have greater body satisfaction, while those with lower self-esteem are more likely to experience body dissatisfaction. Although gender did not emerge as a significant variable influencing self-esteem and body satisfaction within this sample, the limitations related to sample size suggest that further research with larger, more diverse groups is needed to explore potential gender differences more thoroughly.

The complex interplay between Instagram use, body satisfaction, and self-esteem highlights the necessity for comprehensive, multifaceted research approaches. Future research should consider larger sample sizes to enhance the generalizability and statistical power of the findings and ensure balanced gender representation. It is also recommended to incorporate a variety of standardized instruments to increase reliability and validity, include both control (non-Instagram users) and experimental (Instagram users) groups, and investigate specific pathways through which Instagram use might influence the development of eating disorders and body dysmorphic disorders.

Additionally, developing intervention programs that focus on promoting healthy social media use and improving self-esteem and body satisfaction could be beneficial. Future studies should employ both quantitative and qualitative methodologies and consider diverse sample groups to capture a more holistic view of social media’s effects on psychological well-being. These enhancements will contribute to a deeper understanding of the nuanced relationships between social media use, self-esteem, and body satisfaction, ultimately informing more effective prevention and intervention strategies.

## Figures and Tables

**Table 1:** Sociodemographic and descriptive characteristics of the sample (N = 95).

Variable	Frequency (*n*)	Percentage (%)
Educational level		
Compulsory Secondary Education (ESO)	1	1.1%
High School Diploma	4	4.2%
Vocational training (FP)	13	13.7%
University Degree	33	34.7%
Postgraduate/Master’s Degree	33	34.7%
Doctorate/PhD	2	2.1%
Competitive Exam Candidate	9	9.5%
Nationality		
Spain	89	93.7%
France	1	1.1%
Brazil	2	2.1%
Peru	2	2.1%
Germany	1	1.1%
Residence location		
Extremadura	38	39.47%
Comunidad Valenciana	25	26.32%
Madrid	13	13.68%
Salamanca	7	7.37%
Sevilla	4	4.21%
Málaga	3	3.16%
Others	5	5.26%
Profession		
Student	36	37.89%
Psychologist	12	12.63%
Teacher/Professor	9	9.47%
Social Worker	2	2.11%
Veterinarian	3	3.16%
Translator	2	2.11%
Others	31	32.63%
History of eating disorder(s)		
No	89	93.70%
Yes	6	6.30%
Frequency of comparison		
Generally, I don’t do it	54	56.8%
Frequently	13	13.7%
Sometimes	28	29.5%
Time spent thinking about defects		
>1 h	15	15.8%
<1 h	80	84.2%
Purpose of Instagram account		
Leisure	45	47.4%
Personal	40	42.1%
Business	7	7.4%
Other	3	3.2%
Modifications in appearance or lifestyle		
Yes	13	13.7%
No	57	60%
From time to time	25	26.3%

**Table 2:** Statistically significant variables associated with self-esteem and body satisfaction.

	Self-Esteem	Body Satisfaction
Variable	Test and Significance Level	
Profession	H = 17.88 *p* = 0.007 *	
History of ED	U = 135.50 *p* = 0.028 *	U = 106.50 *p* = 0.009 *
Frequency of comparison	H = 22.09 *p* < 0.001 *	H = 17.69 *p* < 0.001 *
Time spent thinking about defects	U = 179.00 *p* < 0.001 *	U = 224.50 *p* < 0.001 *

* The *p*-value indicates statistical significance (*p* < 0.05, CI = 0.95).

**Table 3:** Body satisfaction scores according to BSQ (N = 95).

Body Satisfaction Category	Score Range	Frequency (*n*)	Percentage (%)
High Satisfaction	34–76	41	43.2%
Moderate Satisfaction	77–119	30	31.6%
Low Satisfaction	120–160	19	20.0%
Very Low Satisfaction	161–204	5	5.3%

**Table 4:** Self-esteem scores according to the Rosenberg Self-Esteem Scale (N = 95).

Self-Esteem Category	Score Range	Frequency (*n*)	Percentage (%)
Low Self-Esteem	10–15	20	21.1%
Medium-Low Self-Esteem	16–21	4	4.1%
Medium Self-Esteem	22–27	15	15.8%
High Self-Esteem	28–33	6	6.3%
Very High Self-Esteem	34–40	50	52.6%

## Data Availability

Data are unavailable due to privacy and ethical restrictions.
